# Intravascular Ultrasound (IVUS)-Guided Endovascular Treatment of Severe Left Subclavian Artery Stenosis in a Patient With High-Risk Post-Coronary Artery Bypass Grafting Surgery

**DOI:** 10.7759/cureus.112521

**Published:** 2026-07-12

**Authors:** Abenezer Tedla, Abisiniya Kabtimer, Anshuman Sikka, Elijah Shelby, Charbel Ishak

**Affiliations:** 1 General Surgery, BronxCare Health System, New York City, USA; 2 Surgery, BronxCare Health System, New York City, USA; 3 Interventional Radiology, BronxCare Health System, New York City, USA; 4 Interventional Radiology, Icahn School of Medicine at Mount Sinai, New York City, USA

**Keywords:** balloon-expandable stent graft, coronary subclavian steal, drug-coated balloon angioplasty, hybrid endovascular approach, internal mammary artery graft, ivus guided intervention, myocardial ischemia, subclavian artery stenosis, vertebral artery flow reversal, vertebrobasilar insufficiency

## Abstract

Coronary-subclavian steal syndrome (CSSS) is an uncommon but clinically important entity that may occur after coronary artery bypass grafting (CABG) with internal mammary artery conduits. Hemodynamically significant proximal left subclavian artery (LSA) stenosis can alter distal branch vessel flow and, in patients with prior left internal mammary artery (LIMA)-based CABG, may raise concern for coronary-subclavian steal physiology. Concurrent vertebral artery flow reversal may further predispose to cerebrovascular insufficiency.

We report a case of a 75-year-old male with an extensive cardiovascular history, including prior CABG, transcatheter aortic valve replacement (TAVR), thoracic endovascular aortic repair (TEVAR), and a cardiac implantable electronic device, who presented with dizziness and was found to have severe proximal left subclavian artery stenosis. Computed tomography angiography demonstrated severe (~90%) proximal LSA stenosis, while duplex ultrasonography revealed alternating vertebral artery flow consistent with evolving steal physiology. Invasive hemodynamic assessment confirmed a significant trans-stenotic gradient (>25 mmHg). Given the patient’s prior LIMA-based CABG, severe proximal LSA stenosis raised concern for possible coronary-subclavian steal physiology, although direct angiographic confirmation of retrograde LIMA flow was not available. The patient underwent a hybrid endovascular approach utilizing intravascular ultrasound (IVUS)-guided lesion assessment and optimization, drug-coated balloon (DCB) angioplasty, and precise deployment of a balloon-expandable covered stent (GORE VIABAHN VBX; Flagstaff, AZ: W. L. Gore & Associates). The procedure restored antegrade subclavian flow, normalized the trans-stenotic pressure gradient, and preserved the vertebral artery origin. This case demonstrates the technical feasibility of a multimodality endovascular approach for severe proximal LSA stenosis with evolving vertebrobasilar steal physiology in a high-risk post-CABG patient. Integration of IVUS guidance, DCB therapy, and covered stent technology may assist procedural optimization in carefully selected patients, although further studies are required to define the long-term role of this strategy in supra-aortic trunk disease.

## Introduction

Proximal left subclavian artery (LSA) stenosis represents an important manifestation of systemic atherosclerotic disease with significant cerebrovascular and coronary implications. Hemodynamically significant obstruction can generate pressure gradients sufficient to alter distal branch flow, producing subclavian steal physiology characterized by retrograde or bidirectional vertebral artery flow [1]. While many patients remain asymptomatic, symptomatic cases may present with vertebrobasilar insufficiency, including dizziness, syncope, and neurologic deficits [1].

The clinical significance of proximal LSA disease is markedly amplified in patients with prior coronary artery bypass grafting (CABG) utilizing the left internal mammary artery (LIMA). In this setting, proximal obstruction may result in coronary-subclavian steal syndrome (CSSS), whereby retrograde flow through the LIMA graft diverts blood away from the myocardium to supply the upper extremity [2]. This phenomenon represents functional graft failure despite preserved conduit patency and may manifest as recurrent angina, acute coronary syndrome, or heart failure exacerbation. Reported incidence ranges from approximately 2% to 5% among post-CABG patients, although the true prevalence is likely underestimated [2].

The hemodynamic consequences of proximal LSA stenosis may extend beyond the vertebrobasilar circulation. In the presence of proximal subclavian obstruction, collateralization through reversal of flow in the vertebral and internal mammary arteries may simultaneously compromise both myocardial and cerebral perfusion [1]. In patients with prior LIMA grafting, severe proximal obstruction may raise concern for concomitant coronary hypoperfusion; however, a definitive diagnosis of CSSS requires demonstration of retrograde LIMA flow or objective evidence of myocardial ischemia. Accordingly, careful diagnostic evaluation using clinical assessment and multimodality imaging is essential to define the underlying hemodynamic abnormalities and guide management.

Diagnosis relies on a combination of clinical suspicion and multimodal imaging. Key findings include interarm blood pressure differentials, duplex ultrasonography demonstrating vertebral flow reversal, and confirmation with computed tomography angiography or magnetic resonance angiography [1]. Coronary angiography remains essential for evaluating graft integrity and identifying retrograde flow patterns characteristic of CSSS.

Management strategies have evolved significantly over time. While surgical revascularization, including carotid-subclavian bypass or transposition, offers excellent long-term patency, it is associated with increased perioperative morbidity and prolonged recovery [2,3]. Endovascular therapy has emerged as the preferred first-line approach due to lower procedural morbidity and high technical success rates approaching 97-100% [2,4]. However, proximal ostial LSA lesions near the vertebral artery origin present unique technical challenges, including the need for precise deployment, adequate lesion preparation, and minimization of restenosis risk.

Adjunctive technologies such as intravascular ultrasound (IVUS) have demonstrated improved procedural optimization and long-term patency compared with angiography-guided interventions alone [5]. Drug-coated balloon (DCB) therapy offers a potential strategy to reduce neointimal hyperplasia, while balloon-expandable covered stents provide superior radial strength and deployment precision for ostial lesions [6,7].

In this report, we describe the endovascular management of severe proximal LSA stenosis in a high-risk patient with prior CABG. The intervention incorporated IVUS-guided lesion assessment, adjunctive DCB angioplasty, and precise deployment of a balloon-expandable covered stent to restore subclavian artery patency while preserving the vertebral artery origin. This case highlights the technical feasibility of this multimodality approach while emphasizing the need for further study to define its long-term role in the management of proximal LSA disease.

## Case presentation

A 75-year-old male with a complex cardiovascular history including ischemic cardiomyopathy (left ventricular ejection fraction 40-48%), prior CABG with LIMA conduit, severe aortic stenosis status post-transcatheter aortic valve replacement (TAVR), thoracic endovascular aortic repair (TEVAR), paroxysmal atrial flutter on anticoagulation, implanted dual-chamber automatic implantable cardioverter-defibrillator (AICD) pacemaker, chronic kidney disease stage IV, peripheral arterial disease, hypertension, hyperlipidemia, and insulin-dependent diabetes mellitus presented with a four-day history of dizziness and frontal headache.

He described positional dizziness exacerbated by sitting upright or ambulation. He reported a prior fall before presentation associated with dizziness but denied chest pain, upper extremity claudication, or visual disturbances. On presentation, vital signs were stable. Notably, bilateral upper extremity blood pressure measurements demonstrated asymmetry, with left arm blood pressure measuring 89/51 mmHg compared to 119/57 mmHg in the right arm. Left radial and brachial pulses were diminished (1+), while right-sided pulses were normal (2+).

Neurologic examination revealed preserved cranial nerve function and intact strength in the upper extremities. Mild right lower extremity weakness (3/5) was noted on subsequent evaluation. Laboratory studies were notable for chronic normocytic anemia (hemoglobin 7.4-8.4 g/dL; normal range: 12-16 g/dL), chronic kidney disease (creatinine 1.7-2.3 mg/dL; normal range: 0.5-1.5 mg/dL), and elevated HbA1c (8.2%; normal range: 4.7-6.4%).

**Figure 1:**
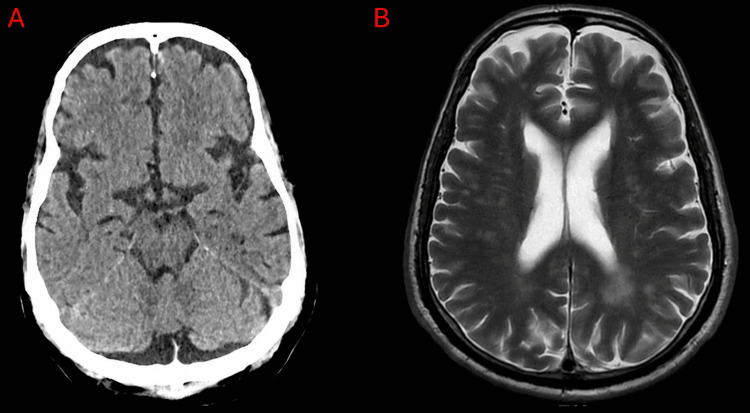
Diagnostic neuroimaging findings. (A) Non-contrast CT head demonstrating chronic microvascular ischemic changes without acute hemorrhage. (B) Brain MRI demonstrating subacute infarction in the parasagittal left posterior frontal lobe.

**Figure 2:**
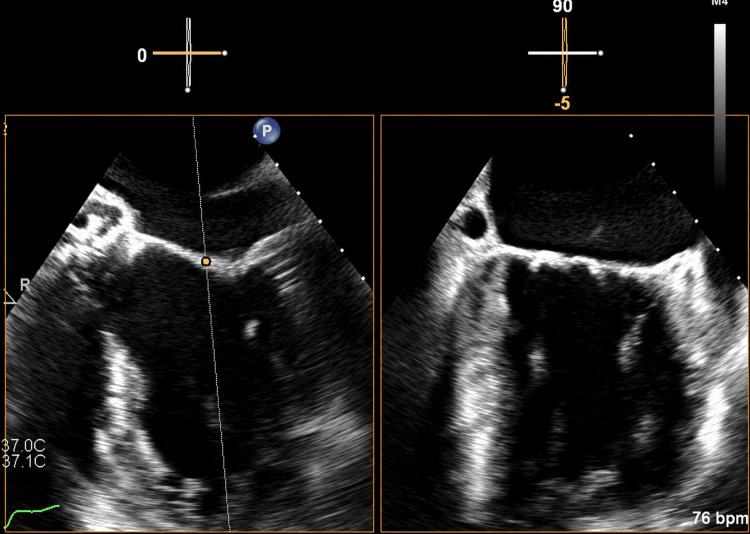
Transesophageal echocardiogram demonstrating an intact interatrial septum without evidence of patent foramen ovale.

Non-contrast CT head on admission demonstrated chronic microvascular ischemic changes without acute hemorrhage (Figure 1A). MRI brain performed during hospitalization revealed a subacute infarction in the parasagittal left posterior frontal lobe within the anterior cerebral artery territory, without hemorrhagic transformation (Figure 1B). Transthoracic and transesophageal echocardiography showed no intracardiac thrombus and no patent foramen ovale (Figure 2).

**Figure 3:**
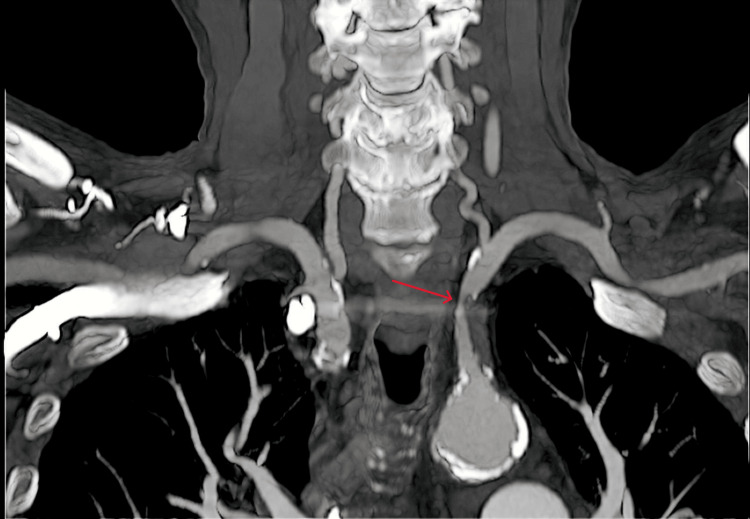
CTA demonstrating severe proximal left subclavian artery stenosis, with the arrow pointing to the area of stenosis (arrow). CTA: computed tomography angiography

Given persistent dizziness and asymmetric upper-extremity blood pressures, further vascular evaluation was pursued. Computed tomography angiography (CTA) of the head and neck performed during hospitalization demonstrated severe (~90%) non-calcified atherosclerotic stenosis at the origin of the left subclavian artery (Figure 3). The residual luminal diameter measured approximately 0.20 cm compared with a reference vessel diameter of approximately 0.90 cm.

**Figure 4:**
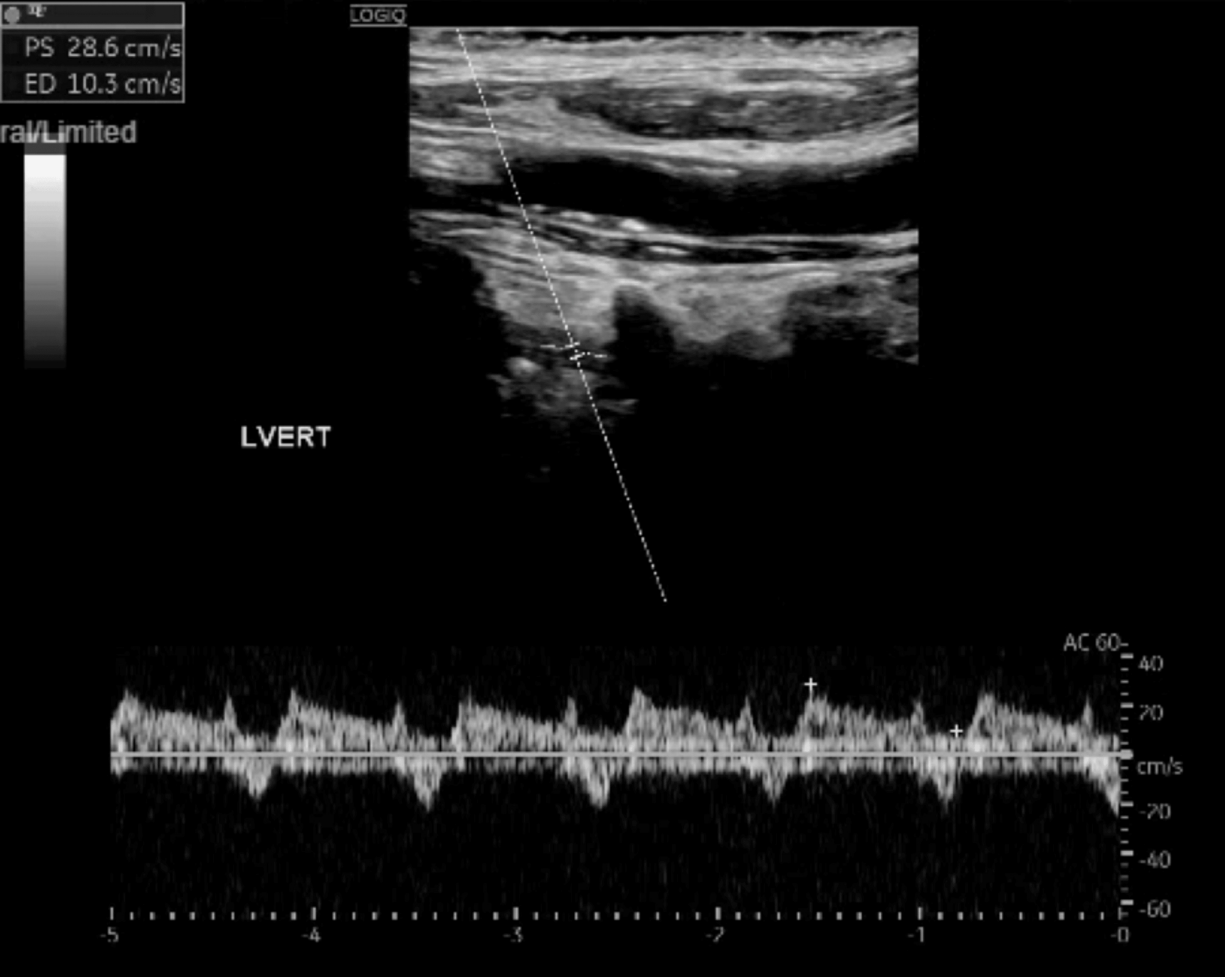
Duplex ultrasound demonstrating bidirectional vertebral artery flow consistent with evolving steal physiology. LVERT: left vertebral artery; PS: peak systolic velocity; ED: end-diastolic velocity; AC: angle correction

Duplex ultrasonography of the extracranial vessels performed during hospitalization demonstrated bilateral internal carotid artery stenosis of 50-69%. The left vertebral artery exhibited alternating (bidirectional) Doppler flow, consistent with evolving subclavian steal physiology (Figure 4). The left subclavian artery waveform demonstrated reduced amplitude. The right vertebral artery flow remained antegrade.

**Figure 5:**
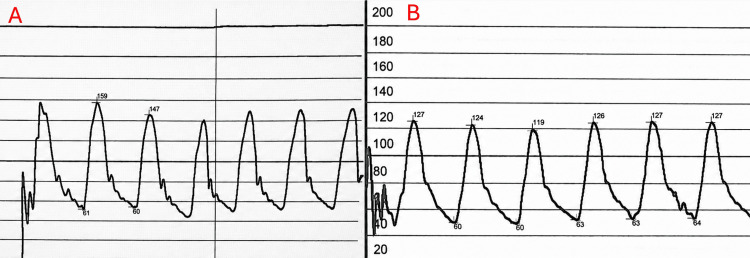
Pre-intervention arterial waveforms in (A) and post-intervention arterial waveforms in (B), demonstrating a trans-stenotic pressure gradient of approximately 25-30 mmHg on angiographic evaluation.

Invasive angiographic evaluation confirmed severe proximal left subclavian artery stenosis with a significant trans-stenotic pressure gradient exceeding 25 mmHg (Figures 5A, 5B). Collateral chest wall circulation was observed. Given the patient’s prior CABG with presumed LIMA graft, concern was raised for coronary-subclavian steal physiology in addition to vertebral flow compromise. Considering the patient’s extensive surgical history, implanted cardiac device, and multiple comorbidities, a decision was made to proceed with endovascular revascularization.

**Figure 6:**
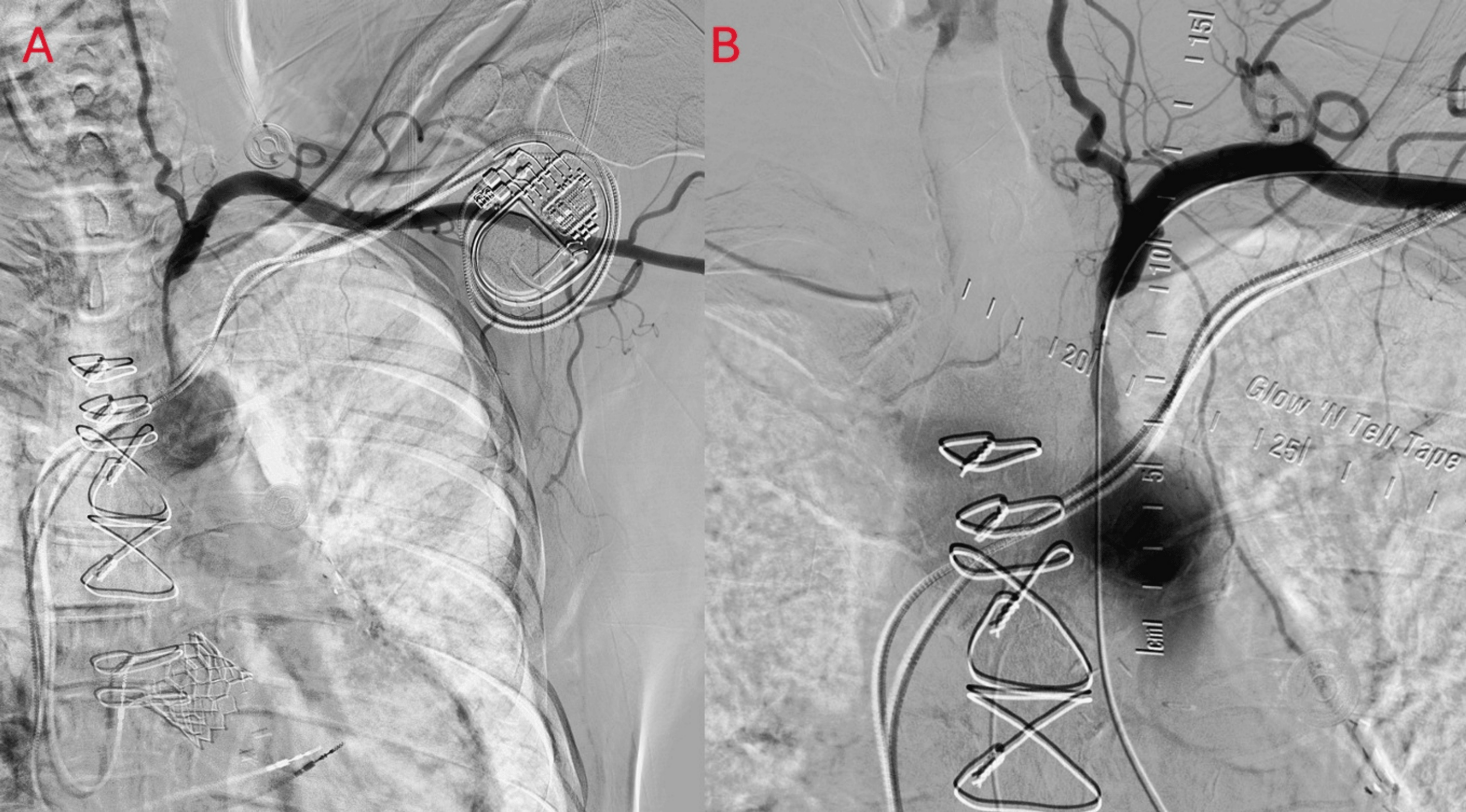
Pre-intervention angiography demonstrating severe ostial LSA stenosis with collateral flow. LSA: left subclavian artery

**Figure 7:**
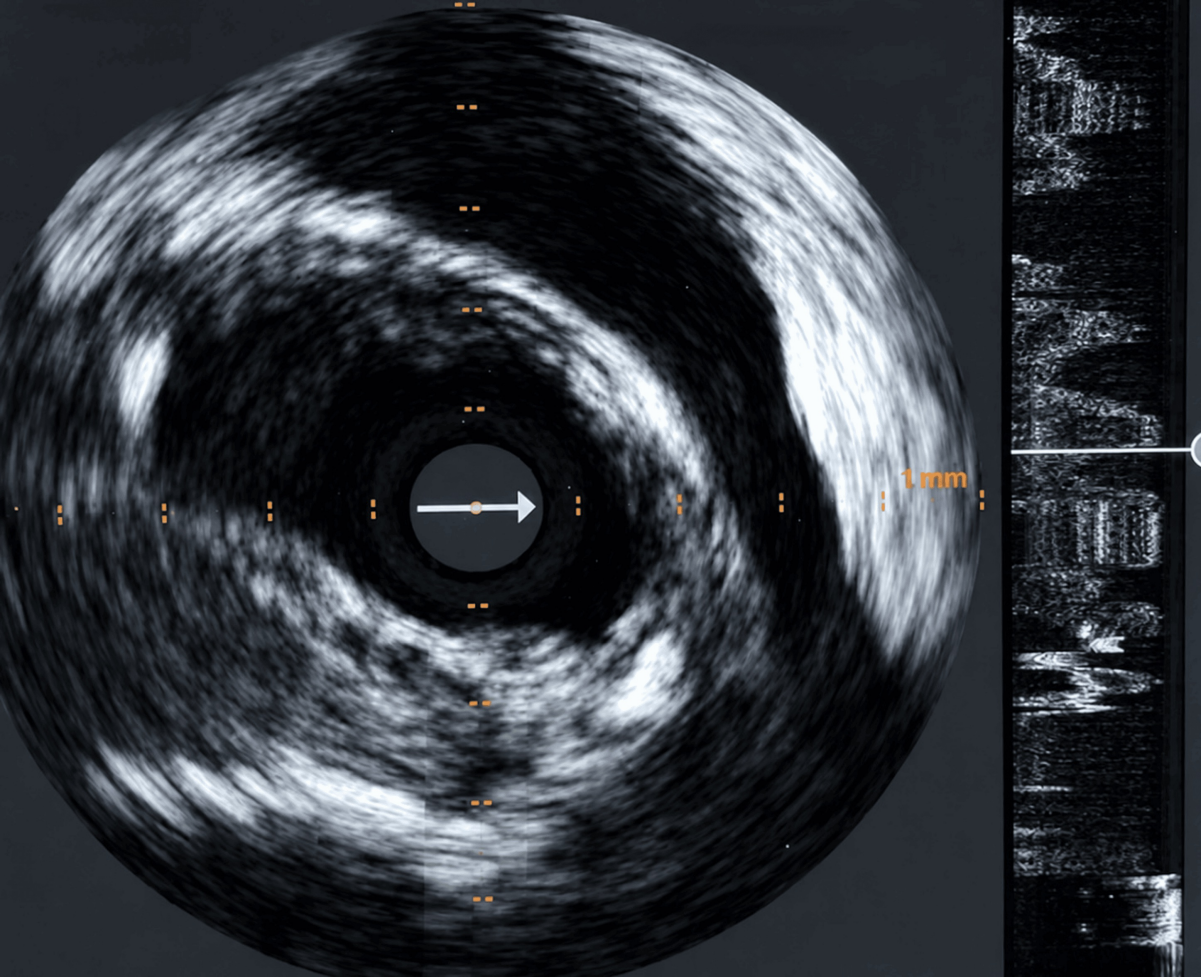
IVUS demonstrating severe concentric plaque and luminal narrowing at the proximal LSA. LSA: left subclavian artery; IVUS: intravascular ultrasound

Subsequently, left brachial arterial access was obtained. Diagnostic angiography confirmed severe ostial left subclavian artery stenosis (Figures 6A, 6B). Intravascular ultrasound (IVUS) (Volcano Eagle Eye Platinum; San Diego, CA: Philips Volcano) demonstrated concentric plaque burden and severe luminal compromise at the proximal segment, with clear visualization of the vertebral artery takeoff (Figure 7).

**Figure 8:**
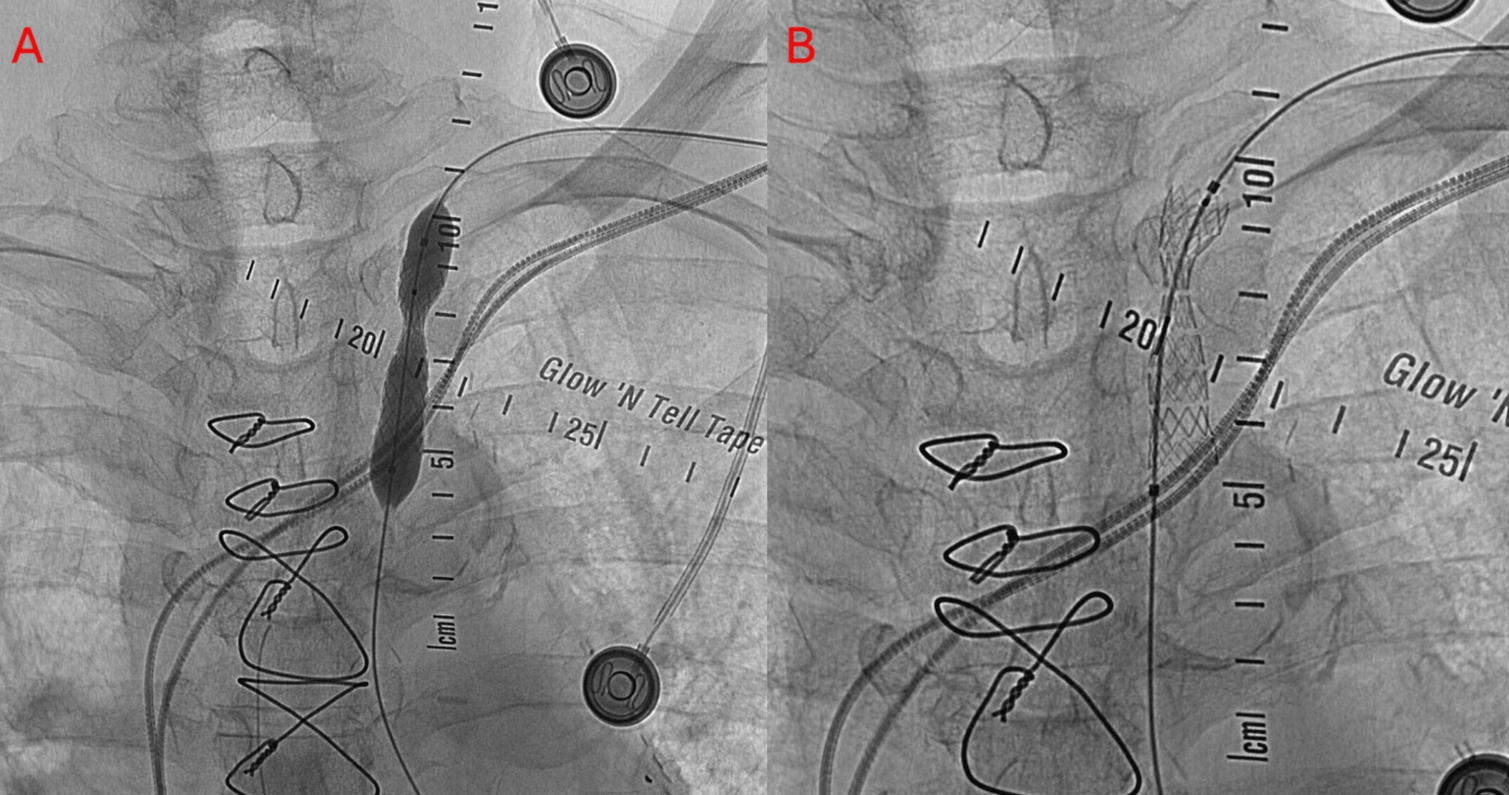
Balloon-expandable stent deployment across proximal LSA lesion. LSA: left subclavian artery

Percutaneous transluminal angioplasty was performed using a drug-coated balloon (BARD Lutonix; Tempe, AZ: Bard Peripheral Vascular, Inc.). This was followed by deployment of a balloon-expandable GORE VIABAHN VBX stent graft (Flagstaff, AZ: W. L. Gore & Associates) across the stenotic segment (Figures 8A, 8B). Careful positioning ensured preservation of the left vertebral artery origin. Post-dilation under IVUS guidance confirmed adequate stent expansion and apposition.

**Figure 9:**
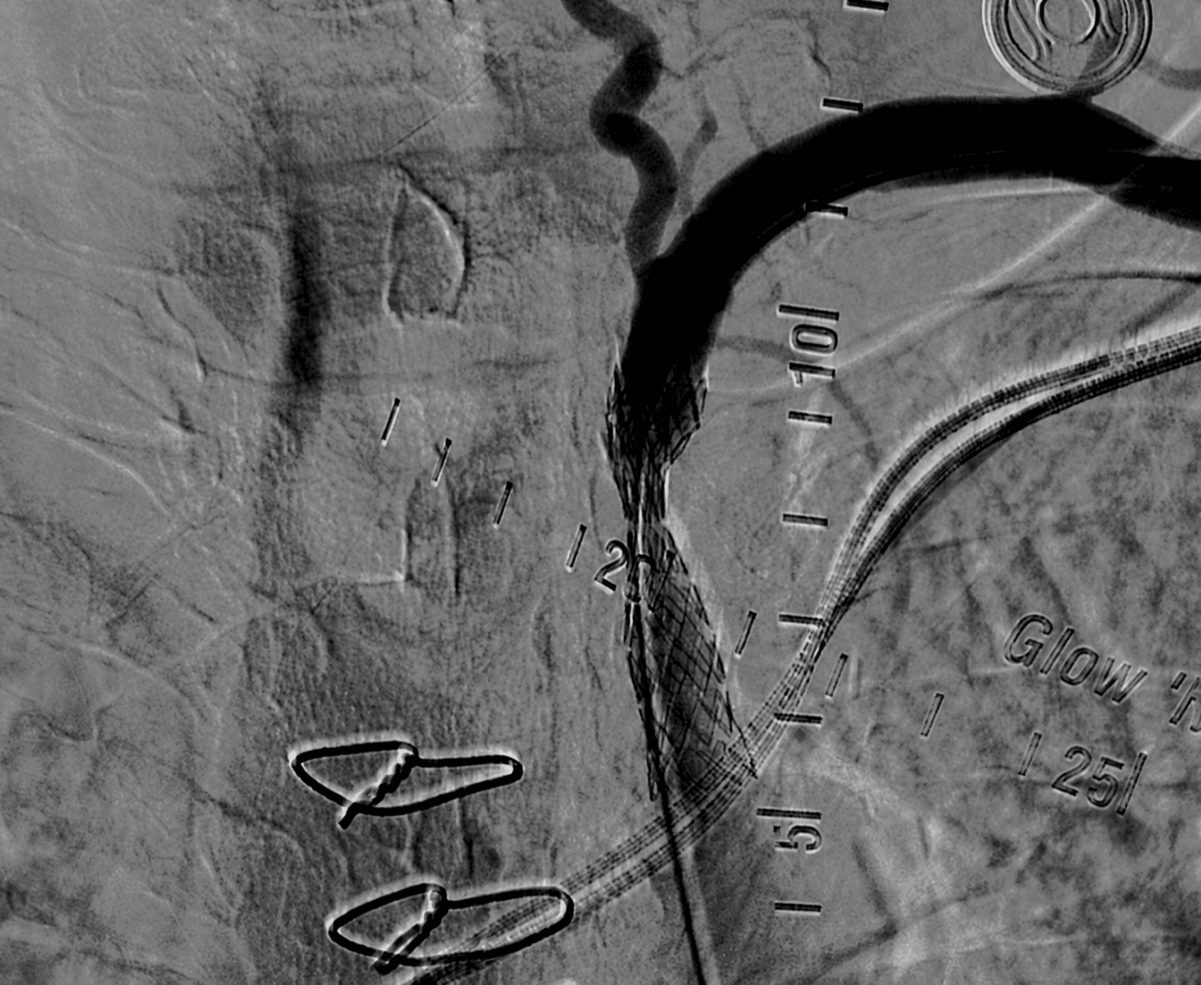
Final angiography demonstrating restored luminal caliber and preserved vertebral artery origin.

Final angiography demonstrated restoration of normal luminal caliber, effacement of collateral chest wall vessels, preserved vertebral artery patency, and normalization of the trans-stenotic pressure gradient (Figure 9). No dissection, embolic complication, or branch occlusion occurred. Hemostasis was achieved with a Terumo Angio-Seal closure device (Somerset, NJ: Terumo Interventional Systems).

The patient tolerated the procedure well. Post-procedural monitoring was uneventful, and no new neurologic deficits were observed. Hemodynamic asymmetry between upper extremities improved following intervention. His dizziness improved during hospitalization, and he was continued on optimized medical therapy for atherosclerotic disease. However, long-term surveillance imaging and extended restenosis assessment were limited due to the relatively short follow-up interval since the procedure.

## Discussion

This case highlights the complex hemodynamic interplay between proximal left subclavian artery (LSA) stenosis, vertebral artery flow alteration, and coronary perfusion in a high-risk post-coronary artery bypass grafting (CABG) patient. Alternating (bidirectional) vertebral artery flow observed on duplex ultrasonography represents an intermediate stage in subclavian steal physiology and is strongly associated with hemodynamically significant proximal subclavian stenosis. Current guidelines from the American College of Cardiology (ACC) and American Heart Association (AHA) state that proximal subclavian obstruction results in reversal of flow in distal branches, including the vertebral and internal mammary arteries, to supply the ipsilateral arm. Interarm blood pressure asymmetry, as demonstrated in this patient, remains a key clinical marker of hemodynamic significance [1].

Although this patient was found to have an anterior cerebral artery (ACA) territory infarction, current evidence does not support a direct causal relationship between isolated proximal left subclavian artery (LSA) stenosis and ACA stroke [8,9]. The infarction was more likely multifactorial, given the presence of atrial flutter, bilateral carotid artery disease, diffuse atherosclerosis, severe anemia, and other vascular comorbidities. Accordingly, proximal LSA stenosis should be regarded as a manifestation of systemic atherosclerotic disease and an independent indication for revascularization because of its hemodynamic significance and potential impact on the upper extremity, vertebrobasilar circulation, and, in patients with prior left internal mammary artery grafting, coronary perfusion.

In patients with prior CABG utilizing a left internal mammary artery (LIMA) graft, severe proximal LSA stenosis should raise clinical suspicion for possible coronary-subclavian steal syndrome (CSSS). CSSS occurs when proximal LSA stenosis results in retrograde flow through the LIMA conduit, diverting blood away from the coronary circulation and potentially leading to myocardial ischemia despite a patent graft. The reported prevalence ranges from approximately 2.5% to 5% in CABG populations, with higher rates in selected cohorts [2,10,11]. Importantly, the absence of angina does not exclude coronary steal, particularly in elderly or functionally limited patients [2,12]. In this context, the identification of severe proximal LSA stenosis in a LIMA-dependent patient carries both diagnostic and therapeutic significance, warranting prompt revascularization to prevent cardiac and neurologic sequelae.

Endovascular therapy has emerged as the preferred first-line treatment for subclavian artery stenosis, particularly in patients with elevated surgical risk. While surgical bypass offers superior long-term primary patency, it is associated with increased perioperative morbidity, including nerve injury and prolonged recovery [2,3]. In contrast, endovascular intervention achieves high technical success rates (>95%) with favorable mid- to long-term outcomes and lower procedural risk [13,14]. Given this patient's extensive comorbidities, including prior CABG, transcatheter aortic valve replacement, thoracic endovascular aortic repair, and ischemic cardiomyopathy, an endovascular approach was both appropriate and guideline-supported [1]. Published evidence regarding endovascular management of subclavian artery disease, including CSSS, IVUS-guided intervention, and adjunctive DCB therapy, is summarized in Table 1.

**Table 1:** Summary of published studies relevant to endovascular treatment of subclavian artery disease and comparison with the current case. CSSS: coronary-subclavian steal syndrome; CABG: coronary artery bypass grafting; PTA: percutaneous transluminal angioplasty; EVT: endovascular therapy; IVUS: intravascular ultrasound; DCB: drug-coated balloon; BMS: bare-metal stent

Studies	Design​	n	Clinical presentation​	Intervention​	Follow-up​	Relevance to current case​
Luo et al. (2026) [15]	RCT	179 ​	Vertebral origin stenosis​	DCB vs. BMS	12 months	Strongest evidence supporting biologic rationale for DCB​
Zhao et al. (2024) [4]	Retrospective cohort	222​	Severe subclavian stenosis	PTA/stenting​	12 month​s	High technical success with acceptable restenosis
Chung et al. (2017)​ [5]	Registry (IVUS analysis)	542​	Subclavian artery disease​	EVT ± IVUS​	5 years​	Higher 5-year patency with IVUS guidance​
Faggioli et al. (2014) [16]	Retrospective case series	10	CSSS after CABG​	PTA ± stents​	15 ± 7 months	Largest dedicated CSSS series supporting endovascular treatment while highlighting procedural risks
This study	Case report​	1	Severe proximal LSA stenosis in a high-risk post-CABG patient	IVUS-guided DCB angioplasty + VBX covered stent	Short-term​	Technical feasibility of combining IVUS, DCB, and covered stent while preserving the vertebral artery origin

A key strength of this case is the use of intravascular ultrasound (IVUS) guidance. IVUS provides superior characterization of lesion morphology, vessel diameter, and stent expansion compared with angiography alone, and its use has been associated with improved long-term patency and reduced adverse events in peripheral interventions [5,17]. Data from the Subclavian Artery Disease Treated With Endovascular Therapy; Multicenter Retrospective (SCALLOP) registry demonstrate significantly higher five-year primary patency with IVUS-guided subclavian interventions compared with angiography alone (88.5% vs. 77.7%) [13,17]. In this case, IVUS enabled precise lesion assessment, optimal device sizing, and confirmation of adequate stent expansion, likely contributing to the favorable procedural outcome.

The adjunctive use of a drug-coated balloon (DCB) prior to covered stent deployment represents a novel aspect of this intervention. Although data in the supra-aortic circulation remain limited, substantial evidence from peripheral, coronary, and neurovascular territories supports the role of DCBs in reducing neointimal hyperplasia and restenosis [6,18,15]. In particular, randomized trials in vertebral and intracranial arteries have demonstrated significantly lower restenosis rates with DCB compared to conventional stenting [18,15]. Although a covered stent ultimately provides the definitive mechanical scaffold, pre-dilation with a DCB was selected based on its antiproliferative mechanism, with the theoretical goal of reducing smooth muscle cell proliferation and neointimal hyperplasia within the treated segment. While extrapolation to the subclavian artery must be done cautiously, the biologic rationale for antiproliferative therapy in large-vessel atherosclerotic disease is compelling. Importantly, contemporary analyses have not demonstrated increased long-term mortality associated with paclitaxel-based devices, supporting their safety profile [19]. Nevertheless, the use of DCBs in the supra-aortic trunk remains off-label, and further studies are required to define their role in this vascular territory.

Preservation of the vertebral artery origin is a critical technical consideration in subclavian interventions. Compromise of vertebral flow may result in posterior circulation ischemia, particularly in patients with contralateral vertebral disease. Various techniques, including embolic protection devices, balloon-guided protection, and dual-balloon strategies, have been described to mitigate this risk [20,21]. In this case, the combination of IVUS guidance, careful stent positioning, and controlled deployment allowed preservation of the vertebral artery without the need for adjunctive protection devices. Restoration of antegrade vertebral flow, preservation of the vertebral artery origin, and normalization of the trans-stenotic pressure gradient demonstrated acute technical success. Long-term clinical benefit and durability remain to be established.

This report has several limitations. As a single case, the findings are not generalizable, and the long-term durability of combined DCB and covered stent therapy in the subclavian artery remains uncertain. Current guidelines acknowledge that the role of drug-eluting technologies in reducing restenosis in subclavian disease has not been definitively established [1]. Additionally, variability among DCB platforms and limited pharmacokinetic data in large-caliber vessels introduce further uncertainty. Prospective studies and dedicated registries are needed to evaluate the safety, efficacy, and durability of this approach.

## Conclusions

Severe proximal left subclavian artery (LSA) stenosis in patients with prior LIMA-based coronary artery bypass grafting (CABG) represents a clinically important lesion because of its potential hemodynamic implications for upper extremity, vertebrobasilar, and coronary perfusion. This case demonstrates the technical feasibility of IVUS-guided endovascular revascularization incorporating adjunctive drug-coated balloon angioplasty and precise covered stent deployment to achieve acute restoration of subclavian artery patency, normalization of the trans-stenotic pressure gradient, and preservation of the vertebral artery origin. Although this multimodality approach was technically successful, the use of adjunctive drug-coated balloon therapy in the supra-aortic circulation remains off-label, and its impact on long-term patency and restenosis is uncertain. Consequently, these findings should be considered hypothesis-generating and require validation in larger studies with long-term follow-up before broader adoption.

## References

[1] Brott TG, Halperin JL, Abbara S (2011). 2011 ASA/ACCF/AHA/AANN/AANS/ACR/ASNR/CNS/SAIP/SCAI/SIR/SNIS/SVM/SVS guideline on the management of patients with extracranial carotid and vertebral artery disease: a report of the American College of Cardiology Foundation/American Heart Association Task Force on Practice Guidelines, and the American Stroke Association, American Association of Neuroscience Nurses, American Association of Neurological Surgeons, American College of Radiology, American Society of Neuroradiology, Congress of Neurological Surgeons, Society of Atherosclerosis Imaging and Prevention, Society for Cardiovascular Angiography and Interventions, Society of Interventional Radiology, Society of NeuroInterventional Surgery, Society for Vascular Medicine, and Society for Vascular Surgery. J Am Coll Cardiol.

[2] Sintek M, Coverstone E, Singh J (2014). Coronary subclavian steal syndrome. Curr Opin Cardiol.

[3] Galyfos GC, Kakisis I, Maltezos C, Geroulakos G (2019). Open versus endovascular treatment of subclavian artery atherosclerotic disease. J Vasc Surg.

[4] Zhao TY, Xu GQ, Xue JY (2024). Effects of percutaneous endovascular angioplasty for severe stenosis or occlusion of subclavian artery. Sci Rep.

[5] Chung WJ, Soga Y, Tomoi Y (2017). Clinical impact of intravascular ultrasound guidance during endovascular treatment of subclavian artery disease. J Endovasc Ther.

[6] Shishehbor MH, Schneider PA, Zeller T (2019). Total IN.PACT drug-coated balloon initiative reporting pooled imaging and propensity-matched cohorts. J Vasc Surg.

[7] Feldman DN, Armstrong EJ, Aronow HD (2020). SCAI guidelines on device selection in aorto-iliac arterial interventions. Catheter Cardiovasc Interv.

[8] Kang SY, Kim JS (2008). Anterior cerebral artery infarction: stroke mechanism and clinical-imaging study in 100 patients. Neurology.

[9] Cho H, Kim T, Kim YD (2022). A clinical study of 288 patients with anterior cerebral artery infarction. J Neurol.

[10] Jabbar AA, Houston J, Burket M, Il'Giovine ZJ, Srivastava BK, Agarwal A (2017). Screening for subclinical subclavian artery stenosis before coronary artery bypass grafting: should we do it?. Echocardiography.

[11] Baudo M, Torregrossa G, Dokollari A (2024). Impact of coronary-subclavian steal after surgical myocardial revascularization with internal thoracic artery in chronic hemodialysis patients: a meta-analysis. Trends Cardiovasc Med.

[12] Papatheodorou N, Argyriou C, Androutsopoulou VA, Chrisafis I, Mikroulis D, Georgiadis GS (2021). Unmasking the coronary-subclavian steal syndrome: the culprit lies in the subclavian artery. a report of a case and review of the literature. Ann Vasc Surg.

[13] Soga Y, Tomoi Y, Fujihara M, Okazaki S, Yamauchi Y, Shintani Y, Suzuki K (2015). Perioperative and long-term outcomes of endovascular treatment for subclavian artery disease from a large multicenter registry. J Endovasc Ther.

[14] Higashimori A, Morioka N, Shiotani S, Fujihara M, Fukuda K, Yokoi Y (2013). Long-term results of primary stenting for subclavian artery disease. Catheter Cardiovasc Interv.

[15] Luo J, Jiang C, Wang H (2026). Effect of drug-coated balloon in patients with severe vertebral artery origin stenosis: a multicenter randomized controlled trial. Int J Stroke.

[16] Faggioli G, Pini R, Cremonesi A (2014). Endovascular treatment of late coronary-subclavian steal syndrome. J Thorac Cardiovasc Surg.

[17] Soga Y, Ariyaratne TV, Secemsky E, Leboucher C, Blein C, Jaff MR, Priest V (2025). Intravascular ultrasound guidance during peripheral vascular interventions: long-term clinical outcomes and costs from the Japanese perspective. J Endovasc Ther.

[18] Guan S, Tong X, Li X (2026). Drug-coated balloon for endovascular treatment of symptomatic intracranial stenotic disease: a multicenter randomized controlled trial. Radiology.

[19] Freisinger E, Koeppe J, Gerss J (2020). Mortality after use of paclitaxel-based devices in peripheral arteries: a real-world safety analysis. Eur Heart J.

[20] Michael TT, Banerjee S, Brilakis E (2009). Subclavian artery intervention with vertebral embolic protection. Catheter Cardiovasc Interv.

[21] Sakamoto S, Abiko M, Kajihara Y (2022). Subclavian artery stenting under cerebral protection by balloon-guiding catheter inflation inside the aortic arch at the left subclavian artery origin. Ann Vasc Surg.

